# Supplementary figures and images for: Accuracy of serological tests for diagnosis of chronic pulmonary aspergillosis: A systematic review and meta-analysis
Source: PLoS One. 2020 Mar 17;15(3):e0222738. doi: 10.1371/journal.pone.0222738 (PMC7077827; doi:10.1371/journal.pone.0222738)

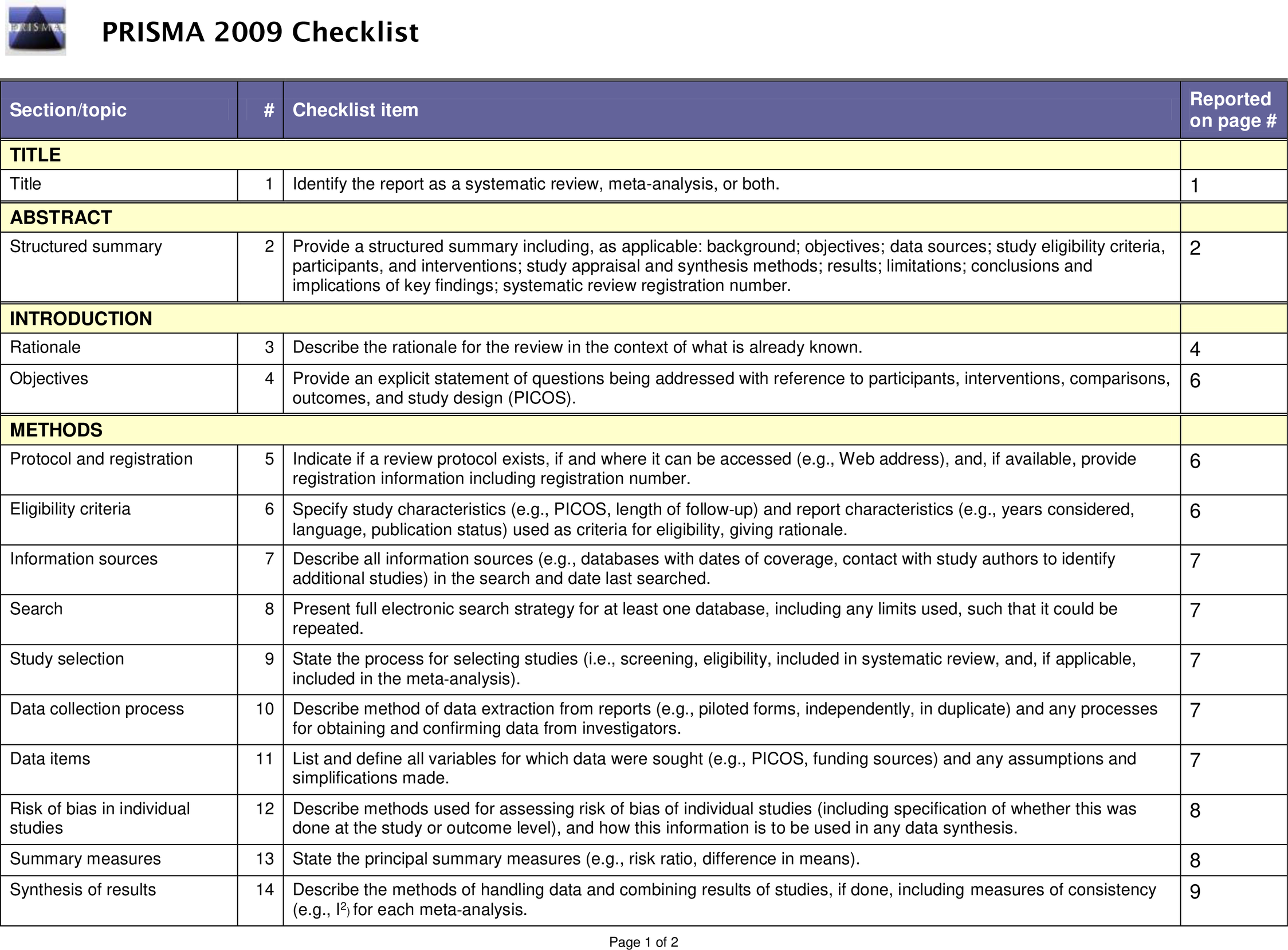

Supplement: S1 Checklist — (TIF) [file pone.0222738.s001.tif]

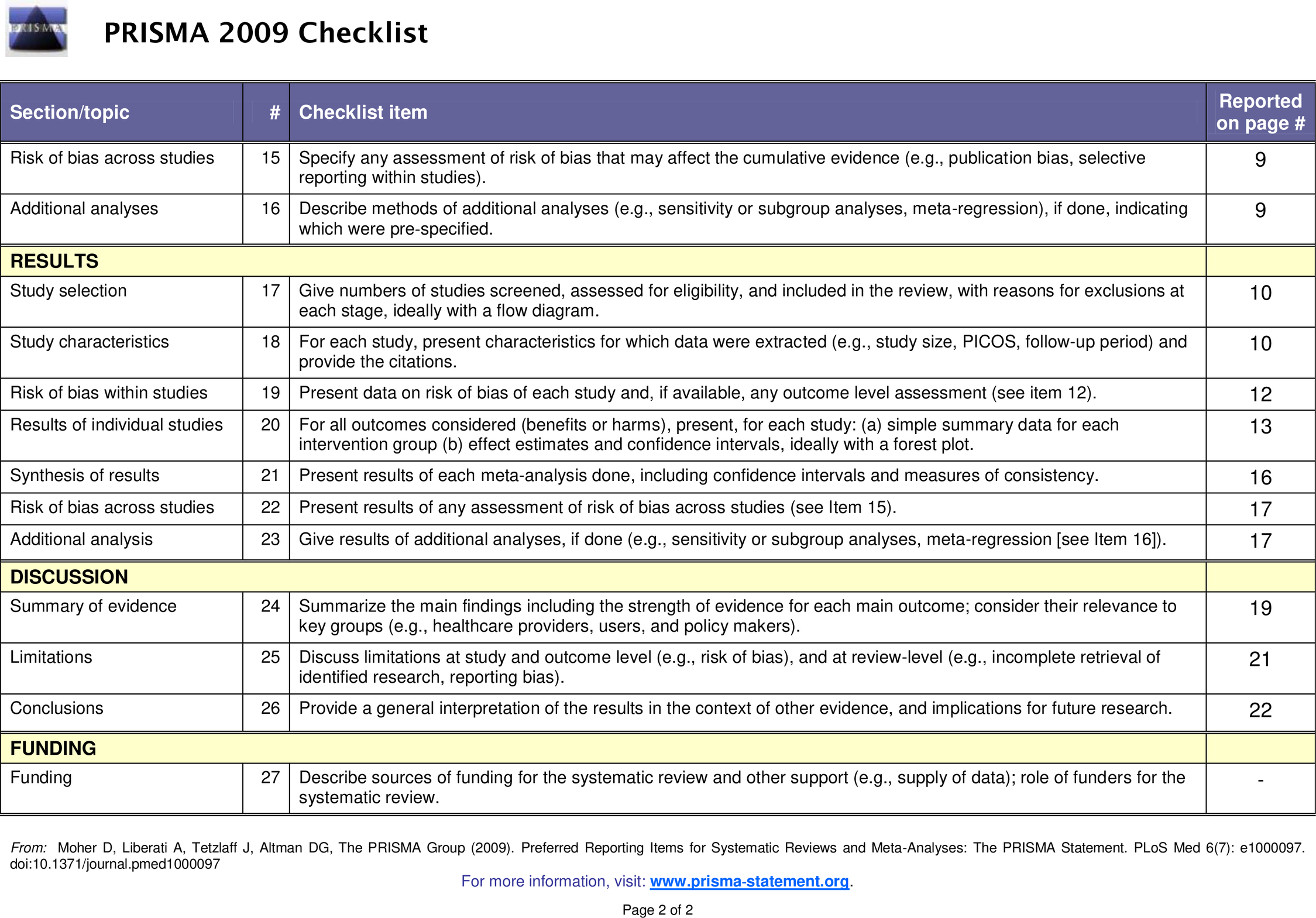

Supplement: S2 Checklist — (TIF) [file pone.0222738.s002.tif]

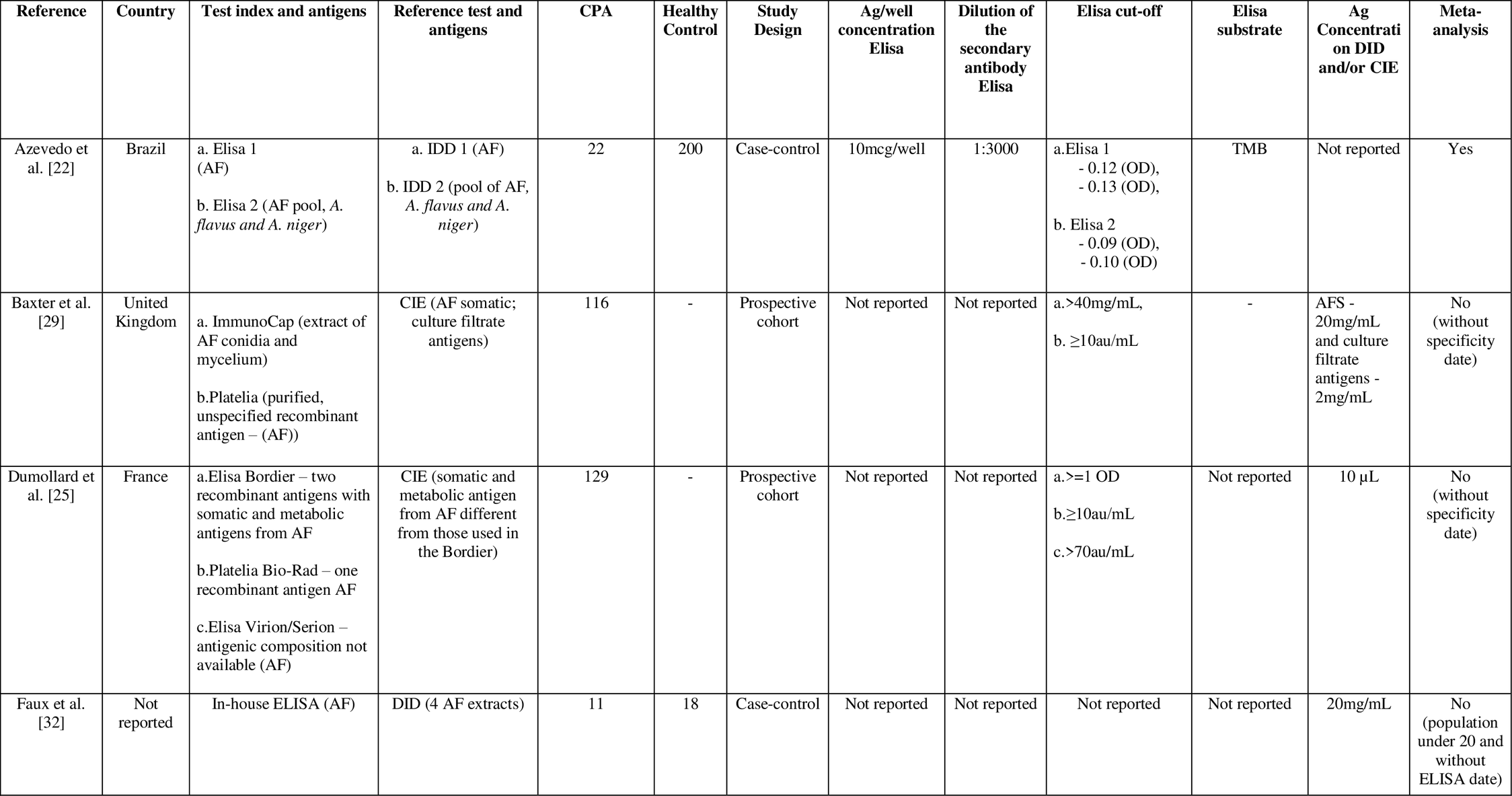

Supplement: S1 Table — ELISA: Enzyme-linked immunosorbent assay; AF: Aspergillus fumigatus; Ag: antigen; DID: double immunodiffusion; CPA: chronic pulmonary aspergillosis; OD: optical density; CIE: counterimmunoelectrophoresis; TMB: 3,3′,5,5′-tetramethylbenzidine; pNPP: alkaline phosphatase yellow; OPD: o-phenylenediamine; RNU: 18 kDa ribonuclease; DPPV: 88 kDa dipeptidylpeptidase; CAT: 360 kDa catalase. (TIF) [file pone.0222738.s003.tif]

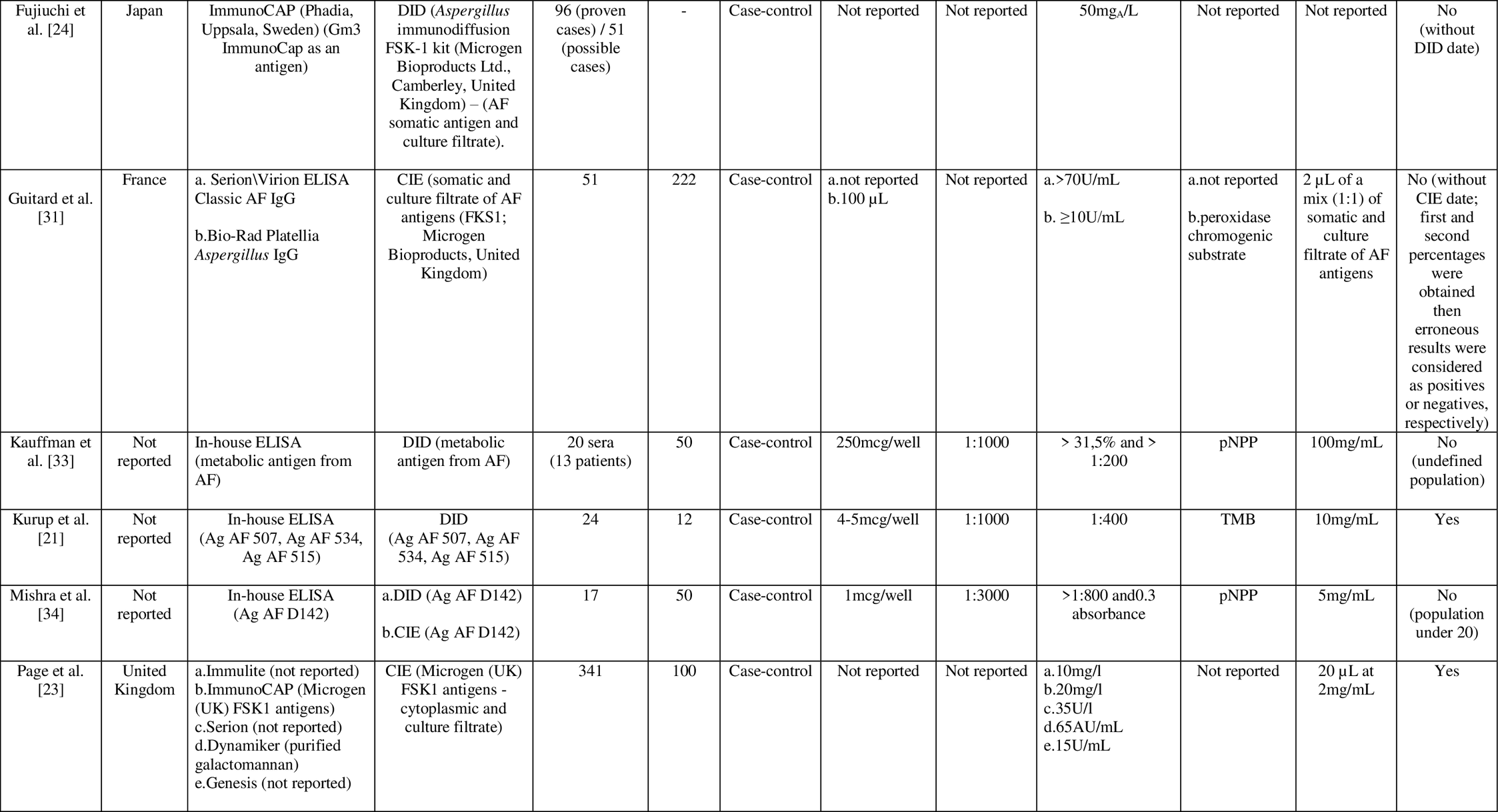

Supplement: S2 Table — (TIF) [file pone.0222738.s004.tif]

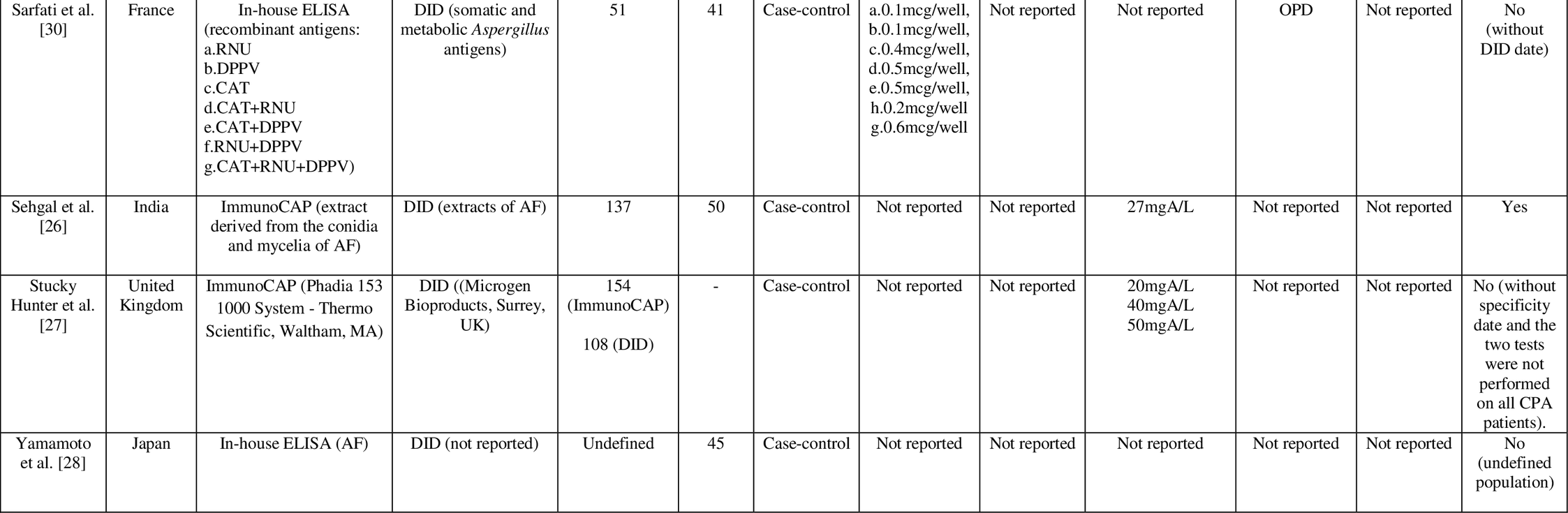

Supplement: S3 Table — (TIF) [file pone.0222738.s005.tif]
