# Supplementary material for: Accuracy of serological tests for diagnosis of chronic pulmonary aspergillosis: A systematic review and meta-analysis
Source: PLoS One. 2020 Mar 17;15(3):e0222738. doi: 10.1371/journal.pone.0222738 (PMC7077827; doi:10.1371/journal.pone.0222738)
Supplement: Attachment — Submitted filename: RESPONSE_TO_REVIEWERS_SYSTEMATIC_REVIEW.doc [file pone.0222738.s006.doc]

**Campo Grande, 20th December 2019.**

**RESPONSE TO REVIEWERS**

**COMMENTS TO AUTHOR: PONE-D-19-24582**

**Reviewer #1:**

Methods:

1) Line 165: The inclusion criteria enlists healthy controls. It could be just written as control group. Sehgal IS have included diseased controls and by this inclusion the study by Sehgal IS et al will be excluded from the review. Generally, diseased controls form a better control group for evaluation of a diagnostic test.

Answer: We changed the inclusion criteria for the control group as suggested in lines 165, 269, and 276 (Table 1). For this reason, we added a subgroup analysis for heterogeneity assessment using only studies with healthy controls in line 171. Results of the subgroup analysis with healthy controls were included in lines 326–329. Figures 7 and 8 were also included for the income statement. We presented the figures in a new sequence for this reason.

2) The authors could include the study by Hunter et al (J Clin Microbiol. 2019 Aug 26;57(9). pii: e00538-19. doi: 10.1128/JCM.00538-19). This study also provides information about ImmunoCap Aspergillus IgG and precipitins in subjects with CPA.

Answer: We welcome the suggestion to include the study by Hunter et al. We included this study for qualitative evaluation as suggested.

3) Could you please discuss about the cut-off values of *A.fumigatus* specific IgG used in different studies?

Answer: We included the discussion of cut-off values in lines 385–389 and the article reference (35) as suggested. We included a column with the cut-off values for the ELISA tests studies evaluated in the review in Table 1.

4) Could you provide a best cut-off for *A.fumigatus*-specific IgG value based on your systematic review?

Answer: Based on individual tests, we included the best ImmunoCAP system cut-off in lines 282–283 and 383–384, as suggested.

5) Please provide reference numbers in the Table 1

Answer: We included the reference numbers in Table 1 and changed the sequence of references throughout the study because we removed two studies and included two other studies based on the suggestions. We also included the reference numbers and made some additional revisions in S1 Table.

6) Please add that diseased controls were used in the study by Sehgal IS, et al. Ref no 27

Answer: We included the use of diseased controls for the study by Sehgal IS et al., lines 212 and 276 (Table 1), as suggested. Given the revisions to the references, we changed the citation number of Sehgal IS et al to [26].

7) Line 351: please correct the spelling of sensitivity

Answer: We corrected the spelling of the word “sensitivity” in line 341.

Discussion:

8) Lines 401-403: This was not the focus of the current systematic review and may be omitted.

Answer: We eliminated the discussion in lines 401–403.

9) Please discuss the short comings of using precipitin tests in discussion. For example these tests are only available at few centers thus are not available to be used for routine practice, even in developing countries. There is lack of standardization of aspergillus antigens that are used across centers. The results of precipitins are semiquantitative and thus do not provide much information.

Answer: We discussed the limitation of precipitin tests in lines 393–396, as suggested, and we have included reference 36.

10) Could you discuss the reason for different cut-off values across different studies? Does it depend on the type of control population used, underlying disease or category of CPA or the ethnicity? For this also use reference Sehgal IS, et al. Efficiency of A. fumigatus-specific IgG and galactomannan testing in the diagnosis of simple aspergilloma. Mycoses. 2019 Aug 13. doi: 10.1111/myc.12987

Answer: We included the discussion of cut-off values in lines 385–389 and the article reference (35), as suggested.

11) What are the clinical implications of the current systematic review?

Answer: We have now included the clinical implications of the study in lines 418–424.

12) Figure 3: Please arrange studies according to the year of publication for better symmetry

Answer: Unfortunately, we cannot arrange studies by year of publication because the Review Manager tool describes the studies in alphabetical order.

13) Figures: The quality of all the figures needs to be improved as they appear to be smudged and are not clear.

Answer: We have produced new figures with better quality for the revised submission.

**Reviewer #2:**

14) The language of the whole manuscript has to be improved by an english native speaker! The first sentence of the abstract (as an example) is no english and hard to read.

Answer: We welcome your suggestion and we will communicate with Editage about the quality of translation into English. We have supplemented the initial translation certificate and the new resubmission certificate. Lines 22–24 of the abstract have now been revised.

15) - Make two sentences out of the first sentence of the introduction.

Answer: We changed the two sentences as suggested.

16) - Site 4, line 54: write only CT scan and delete tomographic images

Answer: We have deleted the words “tomographic images” in line 55 as suggested.

17) - Site 4, line 56: I would not cite reference 5 in this context. Please delete it.

Answer: We deleted reference 5 as suggested and re-arranged the remaining references.

18) - Site 4, line 60: it is not a respiratory tree - please write respiratory tract - correction by an native english speaker is urgently needed.

Answer: We replaced the words “respiratory tree” with “respiratory tract” in line 61.

19) - Site 5, line 85: The epidemiology of *A. fumigatus* is very diverse and differs by region and continents. I would not write it is 40%, because in Europe it is much higher while in Asia it might be lower. So please report the literature correctly.

Answer: We corrected the description in lines 85–87.

20) - To be honest it only makes sence to continue the review process after the manuscript has been improved by an english native speaker otherwise it too hard to read.

Answer: We welcome your suggestion, and we will communicate with Editage about the quality of translation into English. We have supplemented the initial translation certificate and the new resubmission certificate. .

Note: The sentence from lines 77 to 80 has been revised per the request of Dr. van Toorenemberg cited as reference 7 (previous) and current reference 6, by pre-print edition. The previous sentence was “Historically, IgG ELISA assays used in-house antigens, with different antigenic preparations and concentrations, which makes the comparison of test performance very difficult [7].”

Sincerely yours,

Cláudia Elizabeth Volpe Chaves, Msc. Corresponding author.

Phone: +55-67-33782701; Av. Engenheiro Luthero Lopes, n.36 – Bairro Aero Rancho V, Campo Grande, MS, Brazil, CEP 79.084-180. E-mail: [claudiavolpe70@hotmail.com](mailto:claudiavolpe70@hotmail.com) (C.E. Volpe-Chaves).
